# Laparoscopic partial gastric transection and devascularization in order to enhance its flow

**DOI:** 10.1186/1750-1164-2-3

**Published:** 2008-07-07

**Authors:** Federico Cuenca-Abente, Ahmad Assalia, Gianmattia del Genio, Tomasz Rogula, David Nocca, Kazuki Ueda, Michel Gagner

**Affiliations:** 1 Division of Laparoscopic Surgery, Mount Sinai Minimally Invasive Surgery Center (MSMISC), Department of Surgery, Mount Sinai School of Medicine, New York, NY, USA

## Abstract

**Background:**

Esophagogastric fistula following an esophagectomy for cancer is very common. One of the most important factors that leads to its development is gastric isquemia. We hypothesize that laparoscopic gastric devascularization and partial transection is a safe operation that will enhance the vascular flow of the fundus of the stomach.

**Method:**

Our study included eight pigs. Each animal had two operations. In the first one, a laparoscopic gastric devascularization and mobilization took place. Vascular flow was measured previous to the procedure and immediately after it with a laser doppler (endoscopic probe). After three weeks, a second operation took place. We re-measured the vascular flow and sent a sample of gastric fundus for histopathologic evaluation.

**Results:**

The gastric fundus showed signs of neovascularization after both macroscopic and microscopic evaluation. These findings correlated with laser doppler measurements.

**Conclusion:**

Laparoscopic gastric devascularization and partial transection is a safe procedure that increases the vascular flow of the stomach in a three week period. This finding can have a positive impact in terms of decreasing fistula formation.

## 1. Background

Esophagogastric anastomotic leakage is a major cause of morbidity and mortality in patients who underwent an esophagectomy [1-3]. Its incidence varies from 5–25%. Many publications have described the cause of this complication, and it has been described that ischemia of the gastric tube is an important cause [4-6].

Scarce information is available about potential solutions to this problem. Research has been done [7] showing a fall in gastric Pt O2 following gastric devascularization, but not after its mobilization. Some articles [8-10] describe pre-operative embolization as a way of increasing the vascularization of the gastric fundus. Others [11,12] hypothesize surgical devascularization of the gastric fundus and delayed anastomosis as a potential solution. A two stage esophagectomy has been described, but transposing the stomach and making the esophagogastric anastomosis after several days.

In an attempt to increase gastric vascular flow, we performed a gastric devascularization, as done in a standard esophagectomy. Afterwards, the stomach was partially tubulized, making a greater conditioning. Its vascular flow was measured with a laser doppler (PeriMed, Ohio, US) -endoscopic probe- before and after vascular ligature and partial sectioning and, after 3 weeks, a reoperation allowed us to measure vascular flow once again.

Our aim is to evaluate if partial devascularization and transection of the stomach stimulates collateral circulation to develop, and in this way, increase the vascular flow of the gastric fundus.

Additionally, we evaluated the safety of the procedure, in terms of complications, and OR time. The clinical application would be that the risk of esophagogastric anastomotic leakage might be reduced if a better vascularized fundus can be achieved with this technique.

## 2. Methods

Eight animals were used in this study. The pigs were premedicated with IM atropine (0.04 mg/kg) and IM Ketamine (15–25 mg/Kg) or Telazol 5–10 mg/kg IM and received IV Thiopentobarbital (5–11 mg/kg) for induction of anesthesia.

Isofluorane (1.5%) was used for maintenance of anesthesia and titrated to effect. For analgesia, 0.01 mg/kg of IV buprenorphine was administered intra-operatively, and a transdermal Fentanyl patch (100 mcg) was placed for 72 hours post-operatively analgesia. Toradol at 0.3–0.7 mg/kg iv/im or Flunixine meglumine at 0.5–2.2 mg/kg iv/im was used if supplemental analgesia was required.

After induction, an open technique was used to place a 10 mm trocar in the abdomen, and pneumoperithoneum was established.

A 30 degree laparoscope was used. Five 10 mm trocar and one 5 mm trocar were used. As a first step, the vascular flow at the fundus was measured with a laser doppler. With the Harmonic scalpel (Tyco HealthCare, CT, US), the stomach was mobilized (short gastric vessels sectioned). A vascular stapler was used for the transection of the left gastric pedicle.

**Figure 1:**
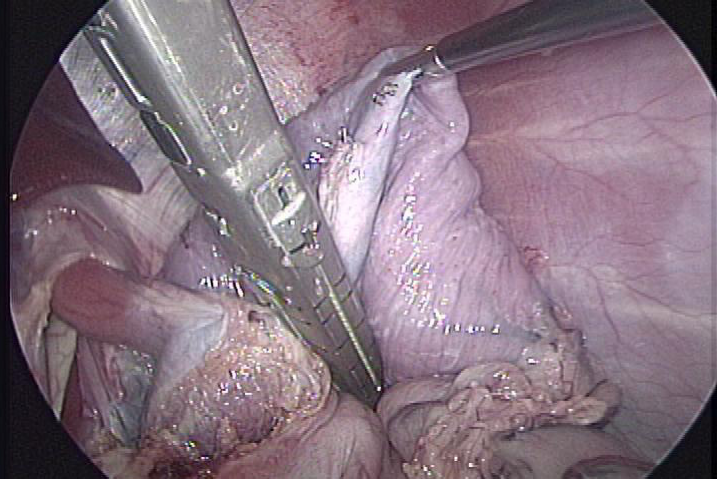
Partial gastric transection after being devascularized.

Following its devascularization, the stomach was be partially tubulized. Beginning from the His's angle, and heading downwards, three sequential cartridges of Endo GIA (Tyco HealthCare, CT, US) were fired [figure 1]. Vascular flow was re-measured at this point.

After the procedure, the animals were maintained NPO for the first 12 hs and then gradually advance from water to solid food over the next 24 hs. Three weeks later, the pigs were re-operated.

The aim of this second procedure was to measure once again the vascular flow at the fundus, and evaluate macroscopically if devascularization had a positive impact in developing collateral vascularization. A sample of gastric fundus was taken for histopathologic evaluation.

## 3. Results

In order to evaluate our results, macroscopic appearance of the fundus after three weeks of the first operation, microscopic evaluation of the fundus after three weeks of the first operation and laser doppler measurements were taken into account.

**Figure 2:**
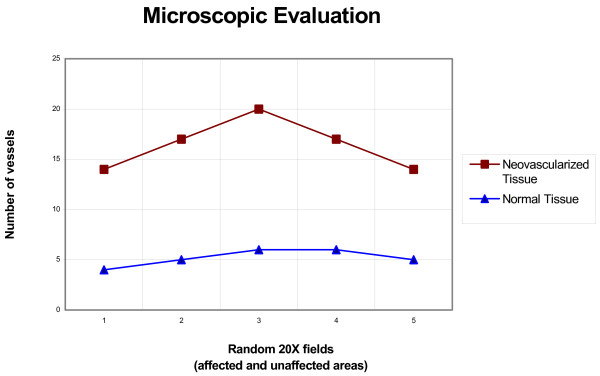
Histopathologic evaluation of gastric tissue. The graphic shows the increase in the number of vessels in the neovascularized portion of the stomach (most distal part of the fundus).

In our series, all the animals had a "pink" appearance during the last operation. This correlates well with the microscopic evaluation, in which an increased number of blood vessels was present in the fundus when compared with histologically normal areas. For this purpose, the vessels (mean value) enclosed within a uniform rectangular area (as defined by a set of reticles within the microscope) in five random 20× fields in the affected and unaffected areas of tissue were measured. The result showed 10/12/14/11/9 and 4/5/6/6/5 vessels respectively [figure 2].

**Figure 3:**
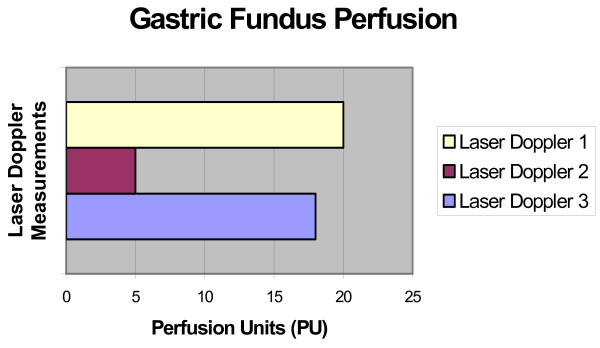
Initial decrease in perfusion after devascularization, and its increase after a three period week.

Laser doppler measurements showed an initial drop in the vascular flow (immediately after the devascularization) and an increase when measured three weeks after [figure 3].

The post operative course of all the animals was uneventful, and the mean OR time was 80 min. No leaks of the stapler line were seen. Ulceration and necrosis, two possible complications of the procedure, were not seen.

## 4. Discusion

Esophagogastric leakage following an esophagectomy in a common cause of morbidity and mortality. Its incidence varies according to the series, ranging from 5% to 25%. In order to decrease fistula rates, many investigators tried to find the source of this problem in order to avoid it. One of the most important factors leading to fistula formation is a low vascular flow, that interferes with the healing process [4-6]. It has also been described that a drop in the vascular flow occurs immediately after its tubulization, and stays stable after its ascension through the thorax up to the neck [7].

Some investigators have performed a gastric devascularization and delayed for 2–3 weeks the anastomosis, finding a better outcome in terms of fistula formation in these animals [11] while others [9,10] used arterial embolization to create a gastric conditioning and applied it in the clinical setting.

The physiologic mechanism of neovascularization development in tissue conditioning has been well described with an analogous procedures. The skin flap transposition for cutaneous reconstruction [13] is an example. In these studies, the flap was partially devascularized and mobilized, and after 2–3 weeks, the skin flap was transposed. Flap necrosis and wound dehiscence were reduced.

In an attempt to decrease the vascularization of the fundus at its best, during the first procedure, we partially transected the stomach as it would be done for a gastric tubulization during an esophagectomy in addition to its devascularization (sectioning of the gastroepiploic, short gastric and left gastric pedicle) and mobilization. In this way, the submucosal vascularization would be reduced, producing a greater ischemic conditioning.

The macroscopic and microscopic examination of the fundus, as well as the measurement of the vascular flow with a laser doppler (PeriMed, Oh, US) with an endoscopic probe, helped us in assessing the effectiveness of the devascularization and neovascularization process.

Potential complications of the procedure we have performed in the series include the ones related to the devascularization and transection processes, and include gastric ulceration and necrosis (devascularization process) and fistula formation secondary to a failure of the stapler line (transection process). None of these complications were present in the ten animals. Besides, though it is not an identical -but similar- procedure, these complications are not widely described in patients operated on for a Collis-Nissen operation.

Regarding the time we took to delay the second procedure, we decided to perform it three weeks after the first one. Our explanation to this relies on previous studies [13] and on the potential clinical application of this "two stage" procedure. One can hypothesize that during the first laparoscopic procedure, the surgeon can perform a diagnostic laparoscopy, the devascularization and partial transection procedure and then, the placement of a feeding jejunostomy. If the patient has metastasis or the tumor is unresectable, no further operation is required. On the other hand, if the patient is suitable for an esophageal resection, a second operation will have to be done. A three week period is a reasonable procedure to feed the patient (most patients with esophageal cancer are malnourished) and allow the stomach to develop neovascularization. Here, another question rises. Is a three weeks period, the top of the curve of the neovascularization process? We do not have that answer, but we think that a three week period is transposable to the clinical setting in both oncological and neovascularization process point of view. As stated before, the first articles reporting conditioning of skin flaps used a 2–3 weeks period, reaching good results in terms of wound healing and avoidance of necrosis [13], and Akiyama et al [9] stated that a minimum period of one week is needed to neovascularize the embolized stomach.

## 5. Conclusion

We can state the this "two stage" procedure can be applicable for cases of esophageal cancer in which the surgeon can offer the patient the minimally invasive approach. If unresectable, the patient will benefit from avoiding a large incision, but if the tumor is amenable to resection, the stomach will have a three week period increase its vascular flow (decrease incidence of fistula) and the surgeon can use this period to feed the patient through the feeding jejunostomy, which is enough to neovascularize the stomach and feed the patient and avoid leaving the tumor in situ and promote its seeding. Further studies will be needed to confirm this hypothesis.
